# The Development of a Social Networking–Based Relatedness Intervention Among Young, First-Time Blood Donors: Pilot Study

**DOI:** 10.2196/publichealth.8972

**Published:** 2018-04-26

**Authors:** Victoria Frye, Louisa Duffy, Janis L France, Debra A Kessler, Mark Rebosa, Beth H Shaz, Bruce W Carlson, Christopher R France

**Affiliations:** ^1^ City University of New York School of Medicine Community Health and Social Medicine City University of New York New York, NY United States; ^2^ New York Blood Center New York, NY United States; ^3^ Ohio University Department of Psychology Athens, OH United States

**Keywords:** blood donation, social media intervention, donor identity, internal motivation

## Abstract

**Background:**

Increasing repeat blood donation behavior is a critical public health goal. According to self-determination theory, the process of developing internal motivation to give blood and an associated self-identity as a blood donor may be promoted by feelings of “relatedness” or a connection to other donors, which may be enhanced through social relations and interactions.

**Objective:**

The purpose of this report it to describe the development and pilot testing of a social networking-based (Facebook) intervention condition designed to increase feelings of relatedness via virtual social interaction and support.

**Methods:**

To develop the intervention condition content, images, text, polls, and video content were assembled. Ohio University college students (N=127) rated the content (82 images/text) presented by computer in random order using a scale of one to five on various dimensions of relatedness. Mean ratings were calculated and analyses of variance were conducted to assess associations among the dimensions. Based on these results, the relatedness intervention was adapted and evaluated for feasibility, acceptability, and preliminary efficacy among 24 first-time donors, aged 18 to 24 years, in a 30-day pilot trial. Paired t-tests were conducted to examine change over time in relatedness and connectedness.

**Results:**

The intervention condition that was developed was acceptable and feasible. Results of the uncontrolled, preintervention, and postintervention evaluation revealed that feelings of individual-level relatedness increased significantly after the intervention.

**Conclusions:**

By promoting first-time blood donor relatedness, our goal is to enhance internal motivation for donating and the integration of the blood donor identity, thus increasing the likelihood of future repeat donation.

**Trial Registration:**

ClinicalTrials.gov NCT02717338; https://clinicaltrials.gov/ct2/show/NCT02717338 (Archived by WebCite at http://www.webcitation.org/6ymHRBCwu)

## Introduction

For decades, research has been conducted to understand repeat blood donation, with the goal of increasing donation efficiency, retention, and diversity of the donor pool. With less than 5% of the US population donating the entire national blood supply, increasing both numbers of blood donors and consistency of donation behavior is critical [1]. Novel approaches and methods are needed to cultivate and grow the core of the blood donor pool: the repeat donor [2]. Repeat donors continue to represent a small group of individuals responsible for donating a significant proportion of the blood in the United States [3]. Repeat donors are particularly critical to the blood supply because they are consistent, committed, and can be counted on to donate in emergencies and under suboptimal conditions. These individuals also tend to be a safer source of blood, for a variety of reasons, and contribute to the overall safety of the blood supply. Finally, in addition to transfusion services needing an appropriate amount of blood, the blood received needs to match the patients’ needs, particularly for sickle cell disease patients. Repeat donors of select blood types and characteristics help transfusion services better meet the clinical needs of the institution’s population, resulting in improved clinical outcomes.

The multi-site, longitudinal National Heart, Lung, and Blood Institute Retrovirus Epidemiology Donor Study (REDS) has followed donors since 1989 and has found four general groups of blood donors: established or multi-year repeat donors; single-year repeat donors; first-time donors; and donors who donated once but never again [4]. Studies of repeat donors have found that they are more often male, white, and with higher education levels [5]; a more recent analysis of donors in a large metropolitan area also found that repeat donors were more likely to be male, white, and older [6]. Although analyses of sociodemographic characteristics are of value, identifying malleable characteristics that act to increase repeat donation behavior is needed to craft effective interventions. Numerous studies have found attitudes, altruism, feelings of social responsibility, self-esteem, self-efficacy, and social pressure to be related to blood donation intention and behavior [7-14]. Donors who give blood more than once within a year of their first donation are associated with long-term donation [15]. Repeat donation varies among different groups of donors; for new donors the intention to donate and age may predict repeat donation behavior, whereas for repeat donors, anticipated regret and a “moral norm” (or feeling of responsibility or duty) may be stronger predictors of repeat donation behavior [16]. Finally, motivation to donate has been found to shift over time for approximately one third of repeat donors, with feelings of solidarity and duty increasing in importance [17]; this suggests the potential that group-based norms and connectedness may influence repeat donor behavior.

Charng and colleagues [18], in their germinal study of donation intentions and behavior, focused attention on social relations and identity as predictors of repeat donation behavior. The authors framed identity as a sense of self that includes various “role identities” which may be established by behavior and reinforced through repetitive or habitual behavior [18]. The importance of the role identity to the sense of self is termed “salience” [19,20]; furthermore, role identity is that part of an individual’s self-concept that derives from membership in a social role or group, along with the attitudes, values, and emotional significance attached to group affiliation [21]. Integrating a role identity as a blood donor into a sense of self may be triggered by an initial positive donation experience and reinforced through repeat donation, which may eventually become habitual. Categorizing oneself as a member of a social group or role may precede the behavior and subsequent repeated behavior [22]. The influence of the social group with which the donor affiliates, and where group members share a group or role identity and engage in social interaction, impacts repeated behavior. Group membership may encourage and reinforce specific behaviors through social interaction, support, connectedness or relatedness, and solidarity [19-21].

A social relations/role identity approach to integrating an identity as a blood donor by increasing feelings of relatedness or connectedness to other donors is also consistent with self-determination theory. Integrating the “blood donor” role into an individual’s identity enhances intrinsic motivation, where autonomous, intrinsically motivated behavior springs from inherent satisfaction/commitment and/or interest [23]. Few interventions have attempted to enhance self-concept or identity as a blood donor by increasing the feeling of connectedness or relatedness to fellow donors through social interaction. Social networking platforms may be ideal vehicles to help develop such interactions and to foster the feelings of relatedness that can encourage donor identity formation. Here we describe the development and assessment of the feasibility, acceptability, and preliminary efficacy of a social network-based (Facebook) intervention condition designed to increase feelings of relatedness among young, first-time blood donors in New York City. This Facebook-based intervention condition was developed and tested in preparation for inclusion as one of four conditions in an ongoing, eight-group, full factorial design, randomized controlled trial of a multi-component intervention to increase blood donation among first-time donors [24].

## Methods

The relatedness intervention condition was developed as a “secret” Facebook group for first-time blood donors. First, we describe the methods used to develop and evaluate the content for the Facebook group; next, we describe methods used to evaluate the preliminary efficacy of the intervention condition on measures of relatedness and social network connectedness. This research was approved by the Institutional Review Boards of New York Blood Center (NYBC) and Ohio University, and registered at ClinicalTrials.gov (NCT02717338).

### Facebook Relatedness Intervention Content Development

To develop the relatedness intervention condition content, we identified images, text, polls, and videos used in previous NYBC social media efforts; novel content, in the form of images and text, was also developed. Each image or text was selected and categorized by the first and second author according to whether they were perceived to increase donor feelings of being supported, respected, valued, connected/belonging, inspired to donate again, or having shared values with other donors. In order to develop the final set of images and text for the intervention, we conducted an evaluation among the focal population of whether the content inspired feelings of relatedness, specifically: belonging to a group, connectedness to other donors, shared values with other donors, and feeling respected by others. Ohio University college students (N=127) rated the content (82 images/text) using a scale of one to five (1 = least positive; 5 = most positive) on the dimensions described above, as well as how much the image was “liked” and was “cool.” Images/text were presented to students via computer and in random order to control for order effects. Mean ratings for each image/text were calculated; analysis of variance (ANOVA) was conducted to assess associations among the rating dimensions. Based on these results, not all of the original 82 images were included in the pilot and some were adapted for use in the pilot study. Highly rated images were included and novel content, designed to encourage social interaction around donation experiences (eg, polls about specific aspects of the most recent donation experience or friends and family who donate), positive identity formation (posts with text/images related to “being a hero” or “saving a life” through donation), and group affiliation and connectedness (logos and visuals specific to the secret Facebook study group that could also be posted to participants’ pages) were developed and included in the pilot trial of the Facebook intervention condition.

### Facebook Relatedness Intervention Condition Pilot

To evaluate the condition’s feasibility, acceptability, and preliminary efficacy, we recruited recent first-time NYBC donors, aged 18 to 24, who agreed to participate in a 30-day pilot study. Invitations to participate were emailed to 95 donors and resulted in an initial recruitment of 24 donors (ie, a response rate of 25%). Donors who agreed to participate completed an online consent form and were invited to join the group for 30 days; they were informed that they would be required to join a “new donor” secret Facebook group. All participants were existing Facebook users. This group was used to expose participants to blood donation-related content and to provide a forum for them to discuss their experiences with blood donation, post images of their donations, and/or describe reasons for donating. Prior to joining the group, participants completed a set of baseline (preintervention) surveys which included basic sociodemographic information and measures of social media connectedness and donor relatedness (described in detail below). To join the secret group, participants were instructed to “friend” the group administrator (the second author), who then sent them an invitation to join the group. Although only current secret group members were able to see posts from the group [25], as an extra safeguard of confidentiality group participants were provided information on how to set their privacy settings prior to joining the group. Participants received a US $30 check if they provided informed consent to participate (n=24), completed the predonation survey (n=24), and joined the Facebook group and completed the post-intervention survey (n=18). The secret group ran for 30 days (SD 7 days) for each participant and was moderated by the second author, who posted content daily and ensured that participant postings conformed to the NYBC policy on acceptable use of social media. Two posts were added each day, with each post typically including an image and text that acted as an invitation for group members to answer a question or poll in an effort to encourage interaction. Once the participant spent 30 days in the group, they were removed from the group and a link to an online follow-up (postintervention) survey was sent to them via email. Paired t-tests were conducted to examine change over time in relatedness and connectedness (see below for a description of the development of the outcome measures). Finally, we assessed acceptability of the Facebook group with three closed-ended items (using true/false response options) that assessed whether participants thought the Facebook group was a good way to connect to other donors or made them want to donate again. We also asked if the participants wished they could have stayed a part of the Facebook group and whether they created a new Facebook account to participate. Finally, one open-ended item was included to generate feedback on how to improve the Facebook group. Responses to this open-ended item were brief and direct, allowing us to summarize the content across all 18 participants.

### Measures

No validated measures existed to assess relatedness or social network connectedness among young blood donors, so we adapted existing measures for this pilot. The donor relatedness measure (Multimedia Appendix 1) was adapted from several sources [26-28]. From the Vlachopoulos and Michailidou scale [28], which was designed to assess relatedness as a subscale of a larger scale to measure autonomy, competence, and relatedness in exercise, we adapted two items reflecting feelings of comfort and friendliness with other blood donors. From the Furrer and Skinner scale [26], originally designed to measure relatedness in a school setting, we adapted one item related to feeling accepted. From Sheldon and Hilpert’s scale [27], originally designed to assess relatedness among Facebook users, we adapted six items reflecting feelings of intimacy and appreciation, as well as aloneness. These nine adapted items were originally included based on their centrality to the construct. We included a single team-developed item, “I feel like a part of a blood donor community.” These 10 items were administered to 923 Ohio University college students with a history of blood donation, using a 7-point response scale (1=not at all; 7=extremely). A principal component factor analysis with varimax rotation was run on the original 10-item scale. Results of this analysis supported a 9-item scale, accounting for 81% of the variance, with items loading on three factors: group-level relatedness (Cronbach α=0.98), individual-level relatedness (Cronbach α=0.86), and nonrelatedness (Cronbach α=0.65). Group-level and individual-level relatedness were positively correlated (r=0.38). Sample items included, “I feel like part of the blood donor community” (group-level relatedness), “I feel a strong sense of intimacy with other blood donors” (individual-level relatedness), and, “I feel alone as a blood donor” (non-relatedness). The social media connectedness scale (Multimedia Appendix 2) was adapted from Grieve et al [29] and tested in the same college student sample. Respondents again rated each item using a 7-point response scale (1=not at all; 7=extremely). Results of a principal components factor analysis varimax rotation supported a final 12-item scale, accounting for 60% of the variance, with items loading on two factors: social media connectedness (Cronbach α=0.87) and social media disconnectedness (Cronbach α=0.85). These scales were negatively correlated (r=-0.42). Sample items included, “I feel close to people on social media” and, “I don’t feel related to most people on social media.”

## Results

### Evaluation of the Relatedness Intervention Content

**Figure 1:**
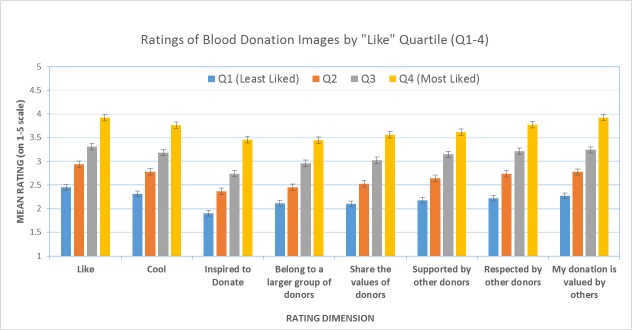
Ratings of images by “like” quartile (N=127).

The student sample (N=127) self-identified as 55% (70/124) women and 45% men (57/127); 42% donors (53/127) and 58% nondonors (74/124). For 80 of the images (80/82, 98%) the “like” ratings did not differ significantly as a function of donor status. As shown in Figure 1, when the images were divided into four groups based on their “like” ratings, ANOVAs revealed that the most liked images were also associated with the most positive responses across each of the other seven domains.

**Figure 2:**
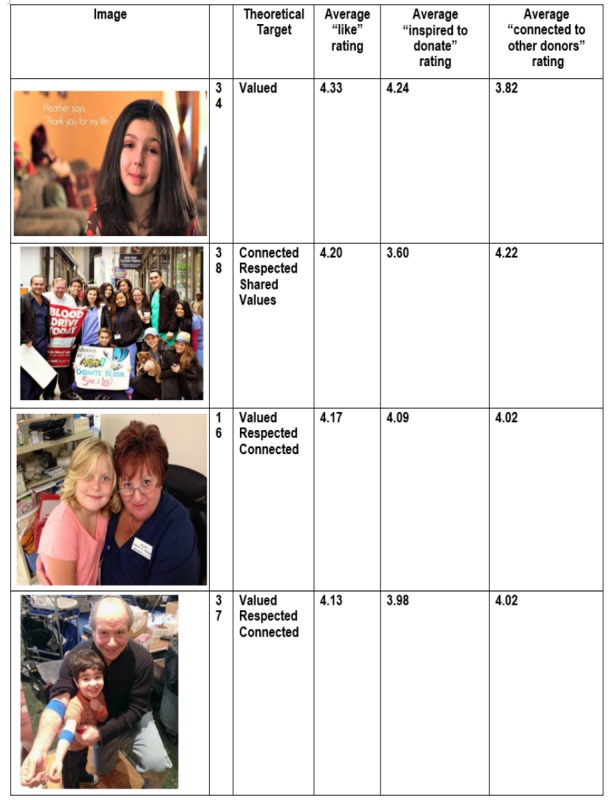
Top five most "liked" images, Facebook relatedness content evauation (N=127).

**Figure 3:**
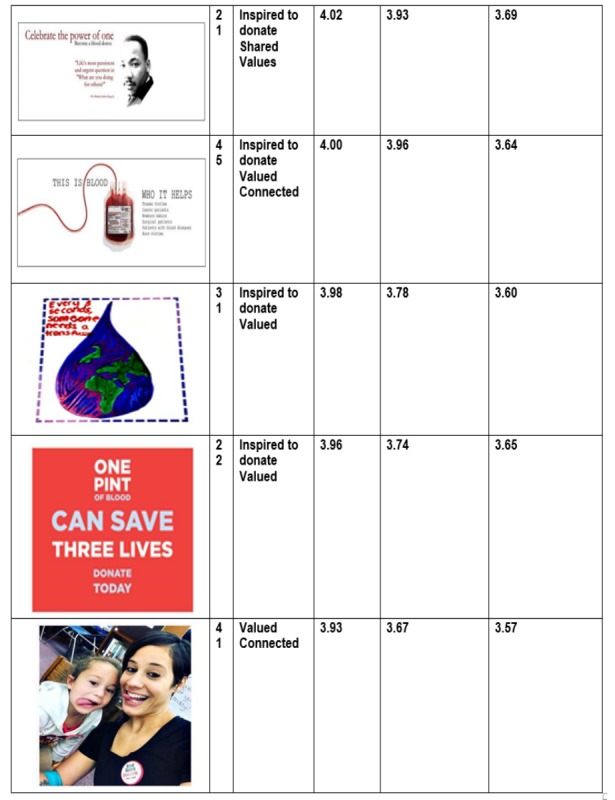
Next five most "liked" images, Facebook relatedness content evauation (N=127).

Examination of the content of the most liked versus least liked images revealed a distinct preference for pictures that conveyed a sense of connection with other people or a direct contribution to the welfare of others. Figure 2 and Figure 3 depict the top ten “most liked” images, the theoretical targets on the various images, as well as the average student ratings by “likes,” “inspired to donate,” and “connectedness.” Student ratings of the same image varied by theoretical target (eg, inspired to donate vs connectedness) although ratings did not vary by donor versus nondonor status. This information informed the selection of images for use in the pilot trial of the relatedness intervention condition.

### Relatedness Condition Intervention Pilot

Of the 24 participants who consented to the study, 75% (18/24) agreed to participate in the relatedness intervention condition pilot trial and completed both the preintervention and postintervention assessments. Interaction with the content was limited, with most posts “liked” by just a few pilot participants. Nonetheless, our analysis revealed a significant increase in feelings of individual-level relatedness from preintervention (mean=3.1, SD=1.2) to postintervention (mean=4.8, SD=1.3; t(17)=5.32, *P*<.001). No significant change was observed for group-level relatedness (*P*=.45) or nonrelatedness (*P*=.64) subscales. There was a marginal increase in social media connectedness from preintervention (mean=3.9, SD=1.3) to postintervention (mean=4.2, SD=1.5; t(17)=1.82, *P*=.09), but no significant change in social media disconnectedness (*P*=.71). We evaluated acceptability using a postintervention assessment. Over three-quarters of participants (14/18, 78%) thought the Facebook group was a good way to connect to other donors and the majority (16/18, 89%) reported that it made them want to donate again. Over half of the respondents (10/18, 56%) reported that they wished they could have stayed a part of the Facebook group. Suggestions to improve the Facebook group included: providing more concrete information on how to donate, actively encouraging group members to share and post, personal messaging to participants, allowing group chats, adding more interactive polls and questions, and adding more participants to the group.

## Discussion

### Principal Findings

In our development and pilot testing of a social network-based relatedness intervention condition for young, first-time blood donors we found that the 30-day membership in a secret and monitored Facebook group was acceptable and feasible. No donors had to create a new account to participate and most continued participation through the end of the 30-day pilot period. In addition, although we did not detect a high volume of interaction among the participants or with the content posted, our pre-post analyses revealed a significant increase in self-reported feelings of blood donor relatedness and a marginally significant increase in social media connectedness. As posited by self-determination theory, an intervention that promotes a sense of belonging or connectedness may aid in satisfying the fundamental human need for relatedness and contribute to a more internalized and self-determined motivation regarding the behavior in question [30]. Thus, we anticipate that this intervention may foster a greater intrinsic motivation regarding blood donation and promote the likelihood of repeat donation behavior.

It is important to note that although we anticipated a postintervention increase in both group- and individual-level relatedness, a significant change was seen only for the individual-level relatedness subscale. Group-level relatedness items tapped feelings of acceptance, comfort, and friendliness with other donors as well as being a part of the blood donor community. Individual-level items reflected feelings of connectedness, intimacy, and contact. Post hoc analyses revealed that a significant change was seen for each item from the individual-level relatedness scale, while no items on the group-level relatedness scale significantly differed from preintervention to postintervention. We may have seen this difference because the social network platform-based intervention acted specifically to increase contact among members thus increasing “individual-level” feelings of relatedness. It is possible that the feelings tapped in the group-level relatedness subscale may change more gradually in response to the intervention than those measured on the individual-level subscale. For instance, if a certain level of individual relatedness is necessary for group-level relatedness to emerge or increase, then we would expect to see a time lag between increases in individual relatedness and group relatedness. If so, the postintervention assessment may have occurred too soon for any lagged group-level relatedness changes to be observed. Further investigation into the observed discrepancy between the intervention effects on group- versus individual-level relatedness is warranted. However, it must also be noted that this is an uncontrolled pilot of a condition and thus caution is urged in interpreting these results.

Another question we will consider in future research using this intervention is whether the level of social interaction in the Facebook group influences the magnitude of change in both individual- and group-level relatedness between participants. As noted, there was limited interaction with the media posted to the secret group page, and yet a significant effect was still seen for individual-level relatedness, along with a marginally significant increase in social media connectedness. A larger sample size, along with the collection of Facebook metrics that were not available in this pilot study, will allow us to evaluate whether a user’s level of engagement with the social network intervention exhibits a dose-response effect on donor relatedness and connectedness.

### Limitations

Although this pilot study provides initial support for the feasibility and acceptability of using a social network platform to increase relatedness among new blood donors, as with any new intervention, this preliminary research has limitations. A potential limitation is that the posts (images and text) were tested among a sample of students in Ohio, whereas the pilot was conducted with donors living in the New York City area. Another potential limitation relates to the lack of a control condition in this pilot trial. In the absence of a suitable control group, it is possible that the positive changes in relatedness were due to demand characteristics and socially desirable responses from participants. Interestingly, the fact that we did not observe the anticipated positive changes in group-level relatedness may be seen as support for the notion that the observed significant effects in individual-level relatedness are not merely an indication of socially desirable responding, as we would expect such an effect across all measures.

An additional limitation regarding the potential efficacy of this intervention relates to whether we can assume that the findings based on a group of donors who volunteer to participate in a Facebook-based study are generalizable to the larger population of donors. Whereas not all donors may be interested or willing to participate in a social network-based intervention, it is reasonable to assume that those who do so may also be the most likely to engage with and benefit from such an approach. Finally, some limitations exist regarding the measures used in this pilot study. While preliminary psychometric testing was conducted in designing our measures of donor relatedness and social connectedness, these scales were developed using a sample of Midwestern college students. Further validation using groups of individuals with greater diversity in age, donation experience, ethnicity, and racial backgrounds is warranted. In addition, as previously mentioned, Facebook metrics that may allow us to gather more data about how participants are interacting with the secret group, and how this may impact relatedness outcomes, were not available in this pilot study. However, this information will be examined as part of an ongoing randomized controlled trial that applies Social Determination Theory to the blood donation context in an effort to enhance retention of first-time blood donors using a combination of donor competence, autonomy, and relatedness interventions [24].

### Conclusions

This pilot study developed and tested the feasibility and preliminary efficacy of a social network platform-based intervention designed to increase a sense of blood donor relatedness among group members. Results of this small, uncontrolled trial suggest that a monitored Facebook group devoted to promoting a blood donor identity may be an effective tool for increasing a feeling of donor relatedness and connectedness among young donors.

## Appendix

Multimedia Appendix 1 Blood Donor Relatedness Scale.

Multimedia Appendix 2 Social Media Connectedness Scale.

## Abbreviations

ANOVA: analysis of variance
NYBC: New York Blood Center
