# Supplementary material for: The Development of a Social Networking–Based Relatedness Intervention Among Young, First-Time Blood Donors: Pilot Study
Source: JMIR Public Health Surveill. 2018 Apr 26;4(2):e44. doi: 10.2196/publichealth.8972 (PMC5945991; doi:10.2196/publichealth.8972)
Supplement: Multimedia Appendix 1 [file publichealth_v4i2e44_app1.pdf]

Blood Donor Relatedness Scale

Directions: When you think about other blood donors, indicate the degree to which you feel each of the following on a scale of 0 ("Not at all") to 6 ("Extremely").		**Not at all**						**Extremely**
1.	I feel accepted by other blood donors.	☐	☐	☐	☐	☐	☐	☐
2.	I feel comfortable with other blood donors.	☐	☐	☐	☐	☐	☐	☐
3.	I feel friendly with other blood donors.	☐	☐	☐	☐	☐	☐	☐
4.	I feel like I am part of a blood donor community.	☐	☐	☐	☐	☐	☐	☐
5.	I feel a sense of contact with other blood donors.	☐	☐	☐	☐	☐	☐	☐
6.	I feel close and connected with other blood donors.	☐	☐	☐	☐	☐	☐	☐
7.	I feel a strong sense of intimacy with other blood donors.	☐	☐	☐	☐	☐	☐	☐
8.	I feel alone as a blood donor.	☐	☐	☐	☐	☐	☐	☐
9.	I have disagreements or conflicts with other blood donors.	☐	☐	☐	☐	☐	☐	☐

Subscale Scores:

- Donor Relatedness, Group Level = Sum of items 1 through 4
- Donor Relatedness, Individual Level = Sum of items 5 through 7
- Donor Non-Relatedness = Sum of items 8 and 9.
