# Supplementary material for: The Development of a Social Networking–Based Relatedness Intervention Among Young, First-Time Blood Donors: Pilot Study
Source: JMIR Public Health Surveill. 2018 Apr 26;4(2):e44. doi: 10.2196/publichealth.8972 (PMC5945991; doi:10.2196/publichealth.8972)
Supplement: Multimedia Appendix 2 [file publichealth_v4i2e44_app2.pdf]

Social Media Connectedness Scale

Directions: Please rate your level of agreement with following statements on a scale of 0 ("Not at all") to 6 ("Extremely").		**Not at all**	___	___	___	___	___	**Extremely**
1.	I feel close to people on social media.	⓪	①	②	③	④	⑤	⑥
2.	I feel understood by the people I know when I'm on social media.	⓪	①	②	③	④	⑤	⑥
3.	My social media friends feel like family.	⓪	①	②	③	④	⑤	⑥
4.	I am able to relate to my social media friends.	⓪	①	②	③	④	⑤	⑥
5.	I find myself actively involved in social media friends' lives.	⓪	①	②	③	④	⑤	⑥
6.	I see social media friends as friendly and approachable.	⓪	①	②	③	④	⑤	⑥
7.	I feel like an outsider when I'm on social media.	⓪	①	②	③	④	⑤	⑥
8.	I see myself as a loner when I am on social media.	⓪	①	②	③	④	⑤	⑥
9.	Even around social media friends I know, I don't feel that I really belong.	⓪	①	②	③	④	⑤	⑥
10.	I feel disconnected from the social media world around me.	⓪	①	②	③	④	⑤	⑥
11.	I don't feel related to most people on social media.	⓪	①	②	③	④	⑤	⑥
12.	I don't feel I participate with anyone or any group on social media.	⓪	①	②	③	④	⑤	⑥

Subscale Scores:

- Social Media Connectedness = Sum of items 1 through 6
- Social Media Disconnectedness = Sum of items 7 through 12
